# Effectiveness of a serious game for medical education on insulin therapy for diabetes: randomized controlled trial

**DOI:** 10.1186/1758-5996-7-S1-A163

**Published:** 2015-11-11

**Authors:** Leandro Arthur Diehhl, Rodrigo Martins de Souza, Pedro Alejandro Gordan, Roberto Zonato Esteves, Izabel Cristina Meister Coelho

**Affiliations:** 1 Universidade Estadual de Londrina (UEL), Londrina, Brazil

## Background

Most patients with diabetes mellitus (DM) are followed by primary care physicians, who often lack knowledge about DM care, especially on insulin. Traditional continuing medical education has little effectiveness, so new educational approaches are required.

## Objectives

To assess the applicability, acceptance, and effectiveness of a serious game for medical education on insulin therapy for DM.

## Methods

A serious game called InsuOnLine© was developed by experts in endocrinology, medical education, and games. Game design was based on modern adult learning theories, and recommendations were adapted from main DM guidelines. A randomized unblinded controlled trial (RCT) was performed to assess the effectiveness of the game, played in “real-world” conditions (in players' own computers and in their free time), compared to a traditional on-site learning activity, both with same content and duration (3-4 h). Primary care physicians from South of Brazil who were not specialists in endocrinology or diabetes were invited to participate, and randomly allocated to one of the groups. Knowledge, problem-solving skills, attitudes, and satisfaction were assessed by a questionnaire applied at baseline, immediately after interventions, and three months later.

## Results

Eighty-eight physicians were allocated to game group; from those, 69 (78%) finished the game, which demonstrates good applicability. Those 69 were included in final analysis. Other 65 physicians were included in control group. Both groups were similar at baseline: 49% were female, and mean age was 38. Both interventions were very well rated, regarding methodology and satisfaction. The gain of knowledge and skills was significant in both groups, with the percentage of right answers going from 52% at baseline to 85% after traditional activity (p<0.001), and to 92% after the game (p<0.001). Three months later, that percentage decreased to 76% in control and to 80% in game group, both it remained significantly higher than at baseline. Absolute increase in performance was higher in the game group (40%) than in control (34%, p=0.01). Attitudes were more significantly improved in the game group than in control. Three months after interventions, all subjects in control and 99% in game group said that the intervention had had real impact on their practice.

## Conclusion

The serious game InsuOnLine© is applicable, well accepted, and very effective for medical education on insulin.

